# The Effect of Obesity and General Anaesthesia Mode on the Frontal QRS-T Angle During Laparoscopic Surgery

**DOI:** 10.3390/diagnostics15151962

**Published:** 2025-08-05

**Authors:** Harun Tolga Duran, Bülent Meriç Çam, Ahmet Salih Tüzen, Muhammet Aydın Akdoğan, Suat Evirgen

**Affiliations:** 1 Department of Anesthesiology and Reanimation, Amasya University Sabuncuoğlu Serefettin Training and Research Hospital, Amasya 05100, Turkey; 2 Department of Anesthesiology and Reanimation, İzmir Katip Çelebi University Atatürk Training and Research Hospital, İzmir 35100, Turkey; astuzen@icloud.com; 3 Department of General Surgery, Amasya University Sabuncuoğlu Serefettin Training and Research Hospital, Amasya 05100, Turkey

**Keywords:** obesity, frontal QRS-T angle, QT interval, anaesthesia

## Abstract

**Background/Objectives**: Obesity is a major cause of repolarisation defects of the heart. The frontal QRS-T angle is a new parameter used for cardiac evaluation. This study aimed to evaluate the effects of a laparoscopic cholecystectomy and anaesthetic agents on the frontal QRS-T angle in individuals with obesity. **Methods**: A total of 91 patients who underwent a laparoscopic cholecystectomy surgery were included in this study. The patients were divided into two groups according to body mass index (BMI) < 30 (*n* = 68) and ≥30 (*n* = 23). The frontal QRS-T angle (FQRST), QT interval (QT), corrected QT, and other electrocardiography (ECG) findings were recorded at different time points. **Results:** In the BMI ≥ 30 group, the frontal QRS-T angle and QT interval measured during the intraoperative period were statistically higher than those of the BMI < 30 group (*p* < 0.001, *p* < 0.001). Additionally, the frontal QRS-T angle value was statistically higher in all patients postoperatively compared with the preoperative and intraoperative periods (*p* < 0.001). Furthermore, there was a positive correlation between the BMI and the frontal QRS-T angle. Our study found that the QRS-T angle and the QT interval duration measured during surgery in the BMI ≥ 30 group who underwent a laparoscopic cholecystectomy were significantly higher than in the BMI < 30 group. **Conclusions**: We recommend close haemodynamic monitoring during and after surgery for patients with obesity undergoing a laparoscopic cholecystectomy.

## 1. Introduction

The increasing prevalence of obesity worldwide brings with it many systemic effects. In addition to improvements in living standards, many lifestyle changes have contributed to rising obesity. An increased body mass index (BMI) is one consequence of morbidity and mortality, and originates from the cardiovascular system. The incidence of obesity has roughly doubled over the last few decades [1]. In particular, a prolonged QT interval related to obesity has been reported [2]. Obesity is a disease characterised by widespread electrocardiography (ECG) disorders. Among these disorders, a prolonged QT interval is frequently observed, which is an indicator of prolonged myocardial repolarisation [3]. Obesity and body fat accumulation cause changes in structural elements. Metabolic effects are very common. Obesity causes prolonged hospitalisation, increased comorbidities, a decreased quality of life, and decreased metabolic health [4,5]. Obesity can cause an increase in morbidity and mortality. It also increases the arrhythmogenic potential. The effect of an increased intra-abdominal pressure in obesity increases the intrathoracic pressure. These changes affect the arrhythmogenic potential. An increased intrathoracic pressure affects the heart, particularly its diastolic function. Diastolic dysfunction can result in restricted myocardial perfusion. Laparoscopic surgeries can trigger these changes [6,7]. Some indications of an arrhythmogenic potential are widely debated. With the widespread use of an ECG for this purpose, different parameters have been considered. As such, the QT interval is widely used as an indicator of the myocardial depolarisation ability. Differences observed in the frontal QRS-T (FQRST) angle between myocardial depolarisation and repolarisation are now widely used as an indicator of myocardial repolarisation [8]. This angle can be measured by subtracting the T axis from the QRS axis on an ECG. The FQRST angle is a useful and easily measurable parameter that is commonly included in ECG reports [9,10].

A successful laparoscopic cholecystectomy reportedly results in less postoperative pain and a shorter hospital stay. However, the formation of a neuroendocrine response due to increased intra-abdominal pressure during a laparoscopic surgery can cause arrhythmia [11,12,13]. The FQRST angle is a reliable indicator of myocardial electrophysiological instability and arrhythmia [14]. Although the effect of the FQRST angle on the QT interval is emphasised, there are few published studies on this topic. In our study, obesity, one of the parameters that may affect the arrhythmogenic potential, was investigated. The hypothesis of the study was that parameters associated with the arrhythmia potential will be observed in obese individuals. In addition to obesity, the effects of an increased intra-abdominal pressure in laparoscopic surgeries were investigated. Our study examined changes in the FQRST angle related to the anaesthesia method used during a laparoscopic cholecystectomy in patients with obesity who underwent the procedure. These effects were analysed alongside changes in the ECG.

## 2. Materials and Methods

### 2.1. Study Design

This prospective observational study was conducted at Amasya University Hospital from 1 January 2025, to 31 May 2025. Ethical approval was received from the Amasya University Ethics Committee with the number 2024000146-2. The study was registered at ClinicalTrials.gov (NCT07034001). We used the CONSORT checklist when writing our report and adhered to the STROBE guidelines.

### 2.2. Patient Selection

A total of 91 patients aged 18–65 years with an American Society of Anesthesiologists (ASA) score of 1–3 who underwent a laparoscopic cholecystectomy between 1 January and 31 May 2025, were included in the study. Patients with known coronary artery disease, atrial fibrillation, heart failure, liver failure, kidney failure, pre-excitation syndromes, electrolyte disorders, respiratory failure, or metabolic diseases and those who were taking drugs that prolong the QT interval were excluded from the study. The ASA risk was determined by the anaesthetist before the study, and the patients’ demographic data (i.e., age, sex, ASA score, and BMI) were recorded. All the participants were informed about the content of the study prior to their participation. It was explained that the anaesthesia method routinely used for patients would be applied. All the included patients provided written informed consent before surgery.

### 2.3. Anaesthesia Management

All the patients were routinely given 500 mL of Ringer’s lactate solution intravenously before anaesthesia and taken to the operating theatre. Their BMI was calculated using the formula body weight/height (kg/m^2^). Patients with obesity had a BMI ≥ 30 kg/m^2^ (group 2) and patients without obesity had a BMI < 30 kg/m^2^ (group 1).

The patients were taken to the operating room, where they were routinely monitored for their ECG results, non-invasive blood pressure, pulse, and oxygen saturation (CARESCAPE™ B650, GE Healthcare, Chicago, IL, USA). All the patients were hydrated intravenously with 500 cc of Ringer’s lactate solution after fasting for 6 h. Midazolam (1 mg IV) and pantoprazole (40 mg IV) were administered as premedication. For anaesthesia induction, propofol (2 mg/kg), fentanyl (2 mg/kg), and rocuronium (0.6 mg/kg) were administered intravenously. Patients without spontaneous breathing were intubated following bag–mask ventilation and connected to a mechanical ventilator. The mechanical ventilation settings were a tidal volume of 6–8 mL/kg, a frequency of 12–15 breaths/min, and a positive end-expiratory pressure of 5–7 cm H_2_O. The anaesthesia was maintained with sevoflurane at 2% in a 50:50 oxygen–air mixture. During the operation, the end-tidal CO_2_ pressure was adjusted to 35 ± 5 mmHg. After anaesthesia preparation was completed, the abdomen was entered with a 10 no trocar and a routine cholecystectomy was completed with an intra-abdominal pressure of 12–14 mmHg and 10 L/min CO_2_ insufflation. After surgery, 200 mg of sugammadex was administered intravenously to terminate the effect of muscle relaxation. Patients with spontaneous breathing were disconnected from ventilator use. Those who were conscious and breathing spontaneously were transferred to the post-anaesthesia care unit. All the patients were monitored with a 12-channel ECG (Cardiofax M ECG-3350, Nihon Kohden, Tokyo, Japan) with a recording speed of 25 mm/s and a resolution of 10 mm/mV. The preoperative recordings were labelled T1, the anaesthesia induction and CO_2_ insufflation were T2, and the postoperative recordings at 6 h were T3. The heart rate, mean arterial pressure, BMI, QT interval, corrected QT interval (QT-c), Tp-e, and FQRST angle values were recorded at all time points.

### 2.4. Electrocardiography

**Figure 1:**
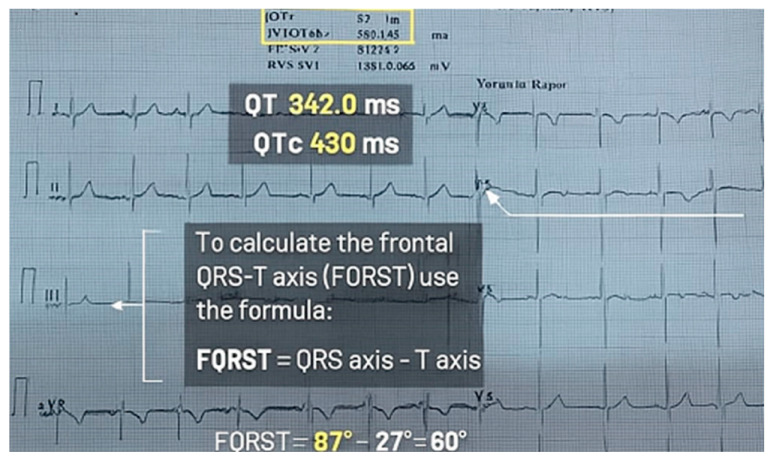
The QT, QT-c, and FQRST angle on the ECG.

Automatic reports were also recorded with the ECG recordings. The QT interval measurements and FQRST angle were taken from the ECG report. The QT and QT-c intervals were measured as previously described [10,15]. The frontal QRS-T angle was calculated as the absolute difference between the QRS axis and the T axis: frontal QRS-T angle = │QRS axis − T axis│ (Figure 1). If the calculated angle exceeded 180°, the angle was subtracted from 360° [12]. This is presented in Figure 1.

### 2.5. Statistical Analyses

A sample study was used to calculate the sample size [16]. The G Power 3.1.9.4 software was used to calculate the patient sample size. The effect size was calculated as 0.505, the type I error margin was 0.05, the power was 0.90, and the total number of patients was 83. To account for possible missing data, we planned to include 91 patients in the study, which exceeds the calculated sample size. The study data were evaluated using the SPSS (Statistical Package for the Social Sciences) software, version 22.0 (SPSS Inc., Chicago, IL, USA). Conformity to a normal distribution was analysed using visual methods (e.g., histograms and probability graphs) and analytical methods (e.g., Kolmogorov–Smirnov test and Shapiro–Wilk test). Parameters that fit a normal distribution were presented as the mean ± standard deviation (SD) and those that did not fit were presented as min–max. For the analysis of two different variables, we used independent sample T-tests and the Mann–Whitney U test. For the analysis of two different parametric tests, a one-way ANOVA was used with a post hoc Bonferroni correction. Pearson’s correlation analysis was used to determine the possible relationship between the BMI and the FQRST angle.

## 3. Results

Of the 91 participants included in the study, 46 (50.5%) were female and there was no statistical difference between the two groups (*p* = 0.918). The number of patients with a BMI ≥ 30 was 23 (25.3%). The mean age of the patients was 47.52 ± 7.21 (mean ± SD) and there was no statistical difference between the two groups (*p* = 0.908). A comparison of the heart rate and mean arterial pressure values at different time periods is presented in Table 1. There was no statistical difference in the QT and QT-c values for the three time periods. However, when the frontal QRS-T angle value was examined, a statistical difference was observed between the groups (*p* = 0.015) A comparison of the HR and MAP values of the patients participating in the study at different time periods is presented in Table 1.

There was no statistical difference in the HR values at the three time periods (*p*: 0.588–0.072–0.078). However, a statistical difference was observed in favour of the group of patients with a BMI ≥30 at all three time periods (*p* < 0.001, *p* < 0.001, *p* < 0.003) (Table 1).

The values of the QT, QT-c, and FQRST angle at different time periods are presented in Table 2.

A comparison of the QT, QT-c, and FQRST angle values of both groups at different time periods is presented in Table 2. In the intraoperative period, it was observed that the patient group with a BMI ≥30 had higher values at all three time points after anaesthesia induction (*p*-values: <0.001, <0.001, and 0.001). There were no statistical differences at the other time points (Table 2).

Table 3 presents a comparison of the QT, QT-c, and FQRST angle values at different time periods.

There were no statistical differences in the QT and QT-c values for the three time periods. However, when the frontal QRS-T angle value was examined, a statistical difference was observed between the groups (*p* = 0.015). The FQRST angle value was not statistically significant in the postoperative period. According to the post hoc Bonferroni correction, the postoperative values were higher than those of the other two time periods (*p* = 0.013–0.007) (Table 3).

**Figure 2:**
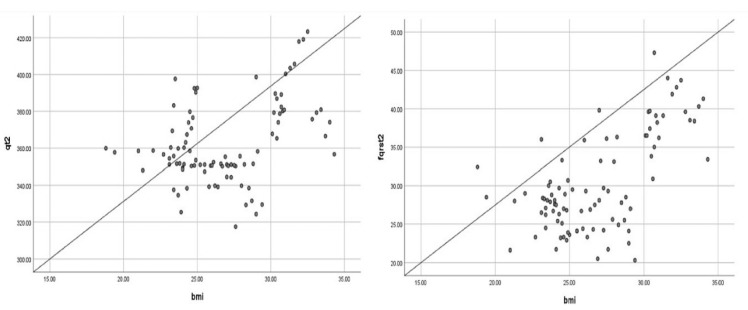
Pearson correlation analysis of frontal QRS-T angle and BMI.

Pearson’s correlation analysis revealed a positive correlation between the BMI and the QT (r = 0.391, *p* < 0.001), QT-c (r = 0.329, *p* = 0.001), and frontal QRS-T angle (r = 0.624, *p* < 0.001) values in the intraoperative period (Figure 2).

A comparison of the Tpe, Tpe/QT, and Tpe/QT-c values in the ECG taken during the intraoperative period in patients with a BMI value of ≥30 is presented in Table 4. The three values taken during the intraoperative period were statistically higher in patients with a BMI ≥ 30 (*p* < 0.001 for all three values). There was no statistical difference between the groups in terms of the other parameters.

Our findings indicated an association between the FQRST angle and individuals with a BMI of ≥30. There were no statistical differences in the heart rate between the two groups at the three different time periods. The FQRST angles of individuals with a BMI < 30 and BMI ≥ 30 were not statistically different in the preoperative period. However, the values in the intraoperative period were statistically higher in individuals. Similarly, the QT and QT-c intervals were higher in individuals with a BMI ≥ 30 during the intraoperative period. There was no statistical difference between the groups in the postoperative period (Table 2). These findings show that the frontal QRS-T angle increased with the induction of anaesthesia in the BMI ≥ 30 group. Pearson’s correlation revealed a positive correlation between the BMI and the FQRST angle value.

## 4. Discussion

Myocardial repolarisation can play a crucial role in the progression of cardiac arrhythmias. During this period, myocardial repolarisation might contribute to arrhythmias caused by cardiac depressant agents. This period on the ECG could be primarily related to the QT and Tp-e durations. Similarly, the FQRST angle measurement is an easily applicable parameter that indicates the arrhythmia potential [16,17]. An increased intra-abdominal pressure can cause an increased intrathoracic pressure, which can lead to systolic dysfunction and an increased arrhythmogenic potential with decreased coronary pressure [18,19].

The FQRST angle varies according to the QRS axis and T wave duration. Studies suggest that changes in age and BMI cause a shift to the left in the cardiac axis, due to an increased fat cell volume in the thorax and changes in the thoracic wall [20,21]. Additionally, the effect of anaesthetic drugs on the FQRST angle remains unclear. It is reported that myocardial repolarisation is affected by sympathetic discharge, particularly during endotracheal intubation [16,22].

Obesity is a clinical condition that causes hypertension and increases systemic vascular resistance. This triggers an increase in the afterload, which can progress to a process that leads to myocardial dysfunction. An increased myocardial workload is a key factor in the development of cardiac arrhythmias. Repolarisation disorders are a chronic consequence of left ventricular hypertrophy and are often accompanied by T-wave abnormalities and a prolonged QRS duration [23,24]. Demir et al. [25] reported a high correlation between the vitamin D level and the FQRST angle in their study. The FQRST angle is considered to be one of the important indicators of sudden cardiac death and morbidity. Uçar et al. [26] reported that the FQRST angle was higher in pregnant women with pre-eclampsia compared to pregnant women without pre-eclampsia. These findings support the hypothesis that an increased FQRST angle is an indicator of cardiac electrophysiological heterogeneity. Several studies indicate that the FQRST angle is a part of cardiac risk assessments. It has been shown to be associated with atrial fibrillation, increased coronary atrial disease, hypertension, and dilated cardiomyopathy [27,28,29,30,31]. This study evaluated the effect of obesity on the FQRST angle. It was concluded that the FQRST angle value increased with the induction of anaesthesia and the beginning of a laparoscopic surgery in obese individuals.

There are few studies in the literature on the relationship between anaesthetic agents and the FQRST angle following endotracheal intubation in patients with obesity [7]. In our study, we concluded that the FQRST angle in patients with obesity was significantly higher during the intraoperative period after anaesthesia induction than at other times. We hypothesise that patients with obesity are at a higher risk of developing a myocardial arrhythmia during the intraoperative period. The intravenous anaesthetic agents used and the increased intra-abdominal pressure during a laparoscopic cholecystectomy surgery might have been effective in this clinical situation. Conversely, the FQRST angle value observed in the postoperative period was higher than at other times for all patient groups. This result could be due to the increased myocardial oxygen demand resulting from increased postoperative pain.

Our study has some limitations. Studies with a large sample size could affect the results. Additionally, examining the ECG record at different time periods might have affected the results. Other limitations of the study include its single-centre design, limited sample size, and lack of long-term follow-up. Furthermore, although an increase in the FQRST angle had arrhythmogenic potential, no cases of malignant arrhythmias were observed among the studied patients.

## 5. Conclusions

In our study, we found that the FQRST angle and the QT interval duration measured during the intraoperative period were significantly higher in patients with a BMI ≥ 30 who underwent a laparoscopic cholecystectomy compared to those with a BMI < 30. Additionally, the FQRST angle value was higher in all patients during the perioperative period. Therefore, we recommend close haemodynamic monitoring during both the intraoperative and postoperative periods for patients with obesity undergoing a laparoscopic cholecystectomy. The assessment of the FQRST angle in anaesthesia practice is important, especially in patients at risk of developing an arrhythmia. It is an easy-to-apply method for identifying patients at risk.

## Figures and Tables

**Table 1:** A comparison of HR and MAP values at different time periods.

	Total	BMI < 30	BMI ≥ 30	*p*
Heart rate				
Preoperative	72 ± 11.6	73.01 ± 12.64	71.47 ± 8.22	0.588 ^a^
Intraoperative	76.69 ± 8.87	75.72 ± 9.61	79.56 ± 5.38	0.072 ^a^
Postoperative	79.24 ± 7.40	77.94 ± 7.23	83.08 ± 6.66	0.078 ^a^
Mean arterial pressure				
Preoperative	91.24 ± 10.09	87.94 ± 8.83	101 ± 6.78	<0.001 ^a^
Intraoperative	89.39 ± 8.82	87.70 ± 8.49	94.39 ± 7.99	0.001 ^a^
Postoperative	85.93 ± 7.60	85.11 ± 7.57	88.34 ± 7.33	0.003 ^a^

^a^ independent sample *t*-test.

**Table 2:** Comparison of QT, QT-c, and FQRST angle measurements at different time periods.

	Total	BMI < 30	BMI ≥ 30	*p*
QT, ms				
Preoperative	356.1 ± 18.48	356.4 ± 17.9	355.16 ± 20.4	0.782 ^a^
Intraoperative	362.2 ± 21.9	354.1 ± 16.78	385.9 ± 17.9	<0.001 ^a^
Postoperative	358.83 ± 16.44	356.3 ± 16.15	363.8 ± 17.7	0.333 ^b^
QT-c, ms				
Preoperative	386.5 ± 18.4	386.9 ± 17.9	385.66 ± 20.42	0.784 ^a^
Intraoperative	387.8 ± 18.78	383.6 ± 16.04	411.6 ± 21.13	<0.001 ^b^
Postoperative	391.1 ± 17.01	389.48 ± 15.67	392.1 ± 20.8	0.527 ^a^
FQRST (°)				
Preoperative	31.48 ± 6.3	31.75 ± 6.48	30.70 ± 5.80	0.495 ^a^
Intraoperative	30.2 ± 6.36	27.36 ± 4	38.83 ± 3.75	<0.001 ^a^
Postoperative	32.96 ± 6.13	33.14 ± 6.67	32.44 ± 4.22	0.559 ^a^

^a^ independent sample *t*-test, ^b^ Mann–Whitney U test.

**Table 3:** Comparison of QT, QT-c, and frontal QRS-T angle values in different time periods.

	Preoperative	Intraoperative	Postoperative	*p*
QT, ms	356.1 ± 18.48	362.2 ± 21.9	358.14 ± 17.03	0.098 ^a^
QT-c, ms	386.5 ± 18.4	389.5 ± 26.2	390.14 ± 17.03	0.472 ^a^
Frontal QRS-T angle (°)	31.48 ± 6.3	30.2 ± 6.36	32.96 ± 6.13	0.015 ^a^

^a^ one-way ANOVA test.

**Table 4:** Comparison of other ECG parameters of the two groups.

	Total	BMI < 30	BMI ≥ 30	*p*
Preoperative				
Tpe	97.02 ± 4.85	96.92 ± 4.19	97.33 ± 6.52	0.728 *
Tpe/QT	0.27 ± 0.02	0.27 ± 0.02	0.27 ± 0.01	0.794 *
Tpe/QT-c	0.25 ± 0.01	0.25 ± 0.01	0.25 ± 0.01	0.791 *
Intraoperative				
Tpe	100.9 ± 10.3	96.03 ± 4.01	115.37 ± 9.74	<0.001 *
Tpe/QT	0.27 ± 0.02	0.27 ± 0.01	0.29 ± 0.02	<0.001 *
Tpe/QT-c	0.25 ± 0.02	0.25 ± 0.02	0.27 ± 0.02	<0.001 *
Postoperative				
Tpe	96.22 ± 4.81	95.6 ± 4.43	98.09 ± 5.48	0.057 *
Tpe/QT	0.26 ± 0.03	0.26 ± 0.03	0.27 ± 0.01	0.124 *
Tpe/QT-c	0.24 ± 0.03	0.24 ± 0.01	0.25 ± 0.01	0.212 *

* independent sample *t*-test, Tpe: T peak to end, QT: QT interval, QT-c: QT-c interval.

## Data Availability

The data produced and/or examined in this study cannot be shared publicly due to patient confidentiality and institutional restrictions, but may be available from the corresponding author upon reasonable request and with permission from Amasya University Training and Research Hospital.
